# Phenotype Switching in Metal-Tolerant Bacteria Isolated from a Hyperaccumulator Plant

**DOI:** 10.3390/biology10090879

**Published:** 2021-09-07

**Authors:** Artur Banach, Agnieszka Kuźniar, Anna Marzec-Grządziel, Anna Gałązka, Agnieszka Wolińska

**Affiliations:** 1 Department of Biology and Biotechnology of Microorganisms, The John Paul II Catholic University of Lublin, Konstantynów St. 1 I, 20-708 Lublin, Poland; agnieszka.kuzniar@kul.pl (A.K.); agnieszka.wolinska@kul.pl (A.W.); 2 Department of Agricultural Microbiology, Institute of Soil Science and Plant Cultivation—State Research Institute, Czartoryskich 8 St., 24-100 Puławy, Poland; agrzadziel@iung.pulawy.pl (A.M.-G.); agalazka@iung.pulawy.pl (A.G.)

**Keywords:** *Azolla*, heavy metals, hydrolytic enzymes, IAA, microbiome, organic pollutants

## Abstract

**Simple Summary:**

*Azolla filiculoides* L. is an aquatic fern with the potential for degradation and accumulation of pollutants. It is accompanied by microorganisms (a microbiome) that may participate in these processes. Microorganisms showing specific phenotypes may promote plant growth in the presence of pollutants. We intended to identify such beneficial strains by studying their potential for the degradation of given organic compounds and the production of hydrolytic enzymes and phytohormones under heavy metal stress (Pb, Cd, Cr (VI), Ni, Ag, and Au). We found 10 isolates displaying varying phenotypes depending on the stress factor. The most efficient was *Delftia* sp., which showed potential for both degradation of organics and plant growth promotion. Other strains were more efficient at metabolizing organics or exhibited enzymatic responses in the presence of the studied metals. These identified phenotypes made all strains beneficial in both supporting plants in unfavorable conditions and degradation of organic compounds. A biopreparation containing these strains may be valuable as both a biofertilizer and a bioremediation agent.

**Abstract:**

As an adaptation to unfavorable conditions, microorganisms may represent different phenotypes. *Azolla filiculoides* L. is a hyperaccumulator of pollutants, but the functions of its microbiome have not been well recognized to date. We aimed to reveal the potential of the microbiome for degradation of organic compounds, as well as its potential to promote plant growth in the presence of heavy metals. We applied the Biolog^TM^ Phenotypic Microarrays platform to study the potential of the microbiome for the degradation of 96 carbon compounds and stress factors and assayed the hydrolytic potential and auxin production by the microorganisms in the presence of Pb, Cd, Cr (VI), Ni, Ag, and Au. We found various phenotype changes depending on the stress factor, suggesting a possible dual function of the studied microorganisms, i.e., in bioremediation and as a biofertilizer for plant growth promotion. *Delftia* sp., *Staphylococcus* sp. and *Microbacterium* sp. exhibited high efficacy in metabolizing organic compounds. *Delftia* sp., *Achromobacter* sp. and *Agrobacterium* sp. were efficient in enzymatic responses and were characterized by metal tolerant. Since each strain exhibited individual phenotype changes due to the studied stresses, they may all be beneficial as both biofertilizers and bioremediation agents, especially when combined in one biopreparation.

## 1. Introduction

Environmental pollution is one of the most important problems that still need to be solved. Numerous types of pollutants are released into the atmosphere, water, and soil, strongly affecting the quality of nature and human life. Therefore, there is still a need to search for new ways to mitigate and remove pollutant loads. Moreover, in order to reduce the human impact on the environment, measures based on natural processes are more desirable than typical techniques that consume resources and which often have negative side effects.

Microorganisms, i.e., the most abundant group of organisms, have many useful traits for coping with stress factors, including contaminants such as heavy metals (HMs) and persistent organics (POP). Nowadays, microorganisms associated with plants are arousing considerable interest. They can be present in the surroundings of plant roots (rhizobacteria) and on the surface (phylosphere) or inside their host (endoshpere). Plant–microbial interactions are a very interesting research subject, as many of them are symbiotic, giving benefits to both partners. Moreover, plants, as primary producers, are essential for life, not only from an ecological point of view, but also as sources of food (agriculture) and useful compounds (e.g., medicinal plants). It is known that microorganisms associated with plants (the so-called plant microbiome) are able to produce many compounds, e.g., phytohormones (auxins or cytokinins), antibiotics, and enzymes (hydrolases and proteases attacking pathogens cells), which ensure plant protection against pathogens and improve plant growth and development. Such microbes are termed Plant Growth-Promoting Bacteria (PGPB) [1,2]. This ability is also observed during plant exposure to contaminants and other stress factors (e.g., temperature, drought, pH); it may help plants to cope with adverse factors or even increase their potential to remove the contaminants. This is referred to as enhanced or assisted phytoremediation [3]. This emphasizes the need to study plant microbiomes to describe their role in supporting the removal of contaminants.

Environmental pressure drives the development of new traits and adaptations to altered conditions, resulting in a switch of microbial metabolisms and the formation of phenotypes adapted to the new situation. New traits have been shown to leave a fingerprint on microbial genomes. These fingerprints can be detected after short-term exposure of bacteria to stress factors by means of phenotypic platforms such as Biolog^TM^ Phenotypic Microarrays (PMs) or simple tests showing changes in microbial enzymatic activity [4]. This information is important for the detection and understanding of changes occurring in the environment, as microorganisms participate in biogeochemical processes and element flow through each environmental compartment. In addition, resistant strains can be identified and used for bioremediation measures [5].

In aquatic ecosystems, HMs exist in many physical states, e.g., dissolved forms (free ions, complex ions, and chelated forms) and particulate forms (colloids, aggregates, precipitates, and nanoparticles) dispersed in water. In addition, they change their state at the sediment–water interface, depending on the physicochemical properties of the system. This makes them more difficult to remove via phytoremediation. HMs can be taken up via roots (soil and water) or leaves (water, air). The response of plants to the presence of metals is regulated via several phytohormones, resulting in triggering defense and response mechanisms, such as promoting the production of antioxidants and chelating compounds. However, the levels of growth stimulating auxin, cytokinins, and gibberellins decrease. In this situation, the presence of microorganisms producing these deficient compounds allows plants to cope with HMs [6,7].

We tested the potential of ten microbial genera (selected as representative genera of the whole epiphytic microbiome) to metabolize different organic contaminants that are often present in the environment and HMs, including those tested previously on *Azolla* sp. [8]. Based on the preliminary work [9], we hypothesized that the microorganisms would be able to synthesize the Indole-3-Acetic Acid (IAA) phytohormone and hydrolytic enzymes, namely cellulases, xylanases, and proteases at exposure to selected doses of metals studied previously. The synthesis of hydrolytic enzymes is extremely important from the point of view of colonization of plant tissues by the studied endophytes [2].

Therefore, the main intention of our study was to provide information about the potential of the microbiome to metabolize organic compounds. We also attempted to evidence its potential to produce plant-supporting substances (IAA, cellulases, xylanases, proteases) during exposure to Pb, Cd, Cr (VI), Ni, Ag, and Au, which may be beneficial for plant-assisted phytoremediation.

## 2. Materials and Methods

Ten previously described and identified strains [9] were selected for the experiment. All strains were selected as representative genera of the identified microbiome of *Azolla filiculoides* (Table 1). Prior to the experiments, fresh cultures on agar medium were prepared from our bacterial collection.

### 2.1. Phenotypic Profile Using Biolog^TM^ GEN III MicroPlates

The 10 pure cultures of strains were identified and characterized using the Biolog GEN III system (Biolog Inc. Hayward, CA, USA), following the manufacturer’s instructions (protocol B). This method allowed the establishment of the metabolic profile for microorganisms, i.e., a “phenotypic fingerprint”. The GEN III MicroPlates™ allowed analysis of bacteria in 94 phenotypic microtests, assessing their ability to metabolize 71 carbon sources. They contained 23 chemical sensitivity assays. All necessary nutrients and biochemicals were prefilled and dried into the 96 wells of the MicroPlate. Tetrazolium redox dyes were used to indicate colorimetrically the use of the carbon sources or resistance to inhibitory chemicals. The isolates were grown on agar medium at the recommended cell density. Then, the cell suspension was inoculated into the GEN III MicroPlate^TM^, 100 μL per well, and the MicroPlate was incubated to allow the phenotypic fingerprint to form. During incubation, there was increased respiration in the wells where cells used a carbon source and/or grew. Increased respiration was responsible for reduction of the tetrazolium redox dye, forming a purple color. Negative wells remained colorless, as did the negative control well (A-1) with no carbon source. There was also a positive control well (A-10) used as a reference for the chemical sensitivity assays in columns 10–12.

Bacterial colonies were transferred to inoculating fluid B (IFB) with a sterile cotton swab to generate bacterial cell suspensions, the transmittance of which was adjusted between 95 and 98% using a turbidimeter (Biolog^TM^). Then, 100 μL of the cell suspension was dispensed into each well. The absorbance of each well of the inoculated microplates was read at 590 nm on a Biolog MicroStation™ at 24-h intervals for five days. The most consistent readings came from 48-h-old Biolog plates, and these data were used in the analyses. Protocol B is used for a small number of strongly reducing species and capsulated species (primarily some strains of *Aeromonas*, *Vibrio*, and spore-forming Gram-positive rods). These species can give a false-positive result in the A-1 well with Protocol A. If this occurs, the test should be repeated using Protocol B.

### 2.2. Metal Tolerance

The tolerance of the microorganisms to Pb, Cd, Cr (VI), Ni, Ag, and Au was tested by determining their minimum inhibitory concentrations (MIC). Each strain was cultured in triplicate on solid nutrient agar medium supplemented with Pb(NO_3_)_2_ (0–1000 mg L^−1^), Cd(NO_3_)_2_ (0–10 mg L^−1^), K_2_Cr_2_O_7_ (0–500 mg L^−1^), NiCl_2_·6H_2_O (0–500 mg L^−1^), AgNO_3_ (0–10 mg L^−1^), and H[AuCl_4_] (0–10 mg L^−1^). Doses showing distinct reduction of bacterial growth after 48 h of incubation were regarded as MIC.

### 2.3. Synthesis of Indole-3-Acetic Acid

The production of IAA in each treatment was conducted by cultivation of the microorganisms on liquid nutrient broth (30 mL) supplemented with 1 g·L^−1^ of L-tryptophan. To this end, we used 24-h cultures with cell densities set to OD_600_ of 1.0. The incubation was conducted in triplicate at 30 °C for 7 days in darkness on a rotary shaker (125 rpm). The samples were collected aseptically after 24, 48, 96, and 168 h. The collected material was centrifuged at 10,000 rpm for 10 min, and 2 mL of the supernatant was mixed with 4 mL of Salkowski’s reagent (50 mL 35% HClO_4_, 1 mL 0.5 M FeCl_3_∙6H_2_O) [10]. After leaving the mixture at 30 °C for 30 min in darkness, the concentration of IAA was measured colorimetrically at 530 nm (Shimadzu UV/VIS-1800, Tokyo, Japan) using a calibration curve up to 100 μg·mL^−1^. The calibration was prepared by processing the IAA solution in the same manner as the samples. The calibration was prepared by processing the IAA solution in the same manner as the samples. The development of a pink color indicated the presence of IAA.

All reagents used were dedicated for microbiological analyses and purchased from Sigma-Aldrich; water was deionized and sterilized before use (sdH_2_O).

### 2.4. Enzymatic Assays

The assays were conducted in a similar manner, i.e., a 24-h strain suspension (30 μL, OD_600_ of 1.0) was poured in triplicate onto a Petri dish with solid nutrient agar medium supplemented with the doses of the tested metals and a substrate characteristic for a given test (test medium). Before pouring the medium on the plates, the pH was set to 7.2.

#### 2.4.1. Cellulolytic Activity

The ability of the strains to produce cellulase was assayed by cultivation of the microorganisms on the test medium supplemented with 1% (*w*/*v*) carboxymethylcellulose sodium salt (CMC) at 30 °C for 48 h. The activity was checked by flooding each plate with Lugol’s solution. The occurrence of clear yellow zones around the colonies against the black background indicated the potential of the strain for degradation of cellulose [9].

#### 2.4.2. Xylanolytic Activity

The xylanase assay consisted of supplementation of the test medium with 1% (*w*/*v*) of xylan. Next, the samples were incubated at 30 °C for 48 h and Lugol’s solution was used to visualize the presence of clear zones around the colonies. The sizes of the zones were recorded and used as a measure of xylanolytic activity [11].

#### 2.4.3. Proteolytic Activity

To detect proteases, the test medium was supplemented with 5% skimmed milk. After 48 h of incubation at 30 °C in the dark, the appearance of clear zones around the colonies indicated proteolytic activity [9].

The sizes of decolorization zones (halos) were used as a measure of given enzymatic activity in all tests.

### 2.5. Statistical Procedures

All experiments were performed in triplicate, except for the Biolog GEN III assays. All relative values are presented as means ± standard deviation (SD). The data from Biolog GEN III experiments were combined in a single matrix, represented as a positive integer, OmniLog^TM^ units (OL units). To illustrate the Biolog results and metabolic profiles, the similarity patterns of the use of the organic compounds and chemical sensitivity assays between all the strains are presented based on individual heatmap graphs. The AWCD of all the strains were calculated, where AWCD was the sum of the differences between the OL units of the blank well (water) and substrate wells divided by 95 (the number of substrate wells in the GEN III MicroPlates^TM^), after 48 h of incubation. For the substrates, Shannon diversity (H’) and Shannon evenness (E) indices were also assessed. A multivariate statistical method using PCA was performed to summarize the variability of the tested strains and to determine the association among the measured activities. For Spearman’s rank correlation, PCA, and heatmap analysis, all the data were standardized so that each score contributed equally to the analysis. MANOVA was used to test the effect of the metals and their doses on bacterial enzymatic activity and phytohormone synthesis. One-way ANOVA was used to check the significance of the differences of AWCD for each group of compounds/conditions for the strains. For both tests, Tukey’s post hoc procedure was used to assess the differences between the groups. Normality and equality of variances were examined using Shapiro–Wilk and Levene’s statistics, respectively. The significance was assumed at *p* < 0.05. All these analyses were performed using Statistica 13.1 (StatSoft. Inc., Tulsa, OK, USA).

## 3. Results

### 3.1. Phenotypic Characterization of Bacteria Using BiologTM GEN III MicroPlates

**Figure 1:**
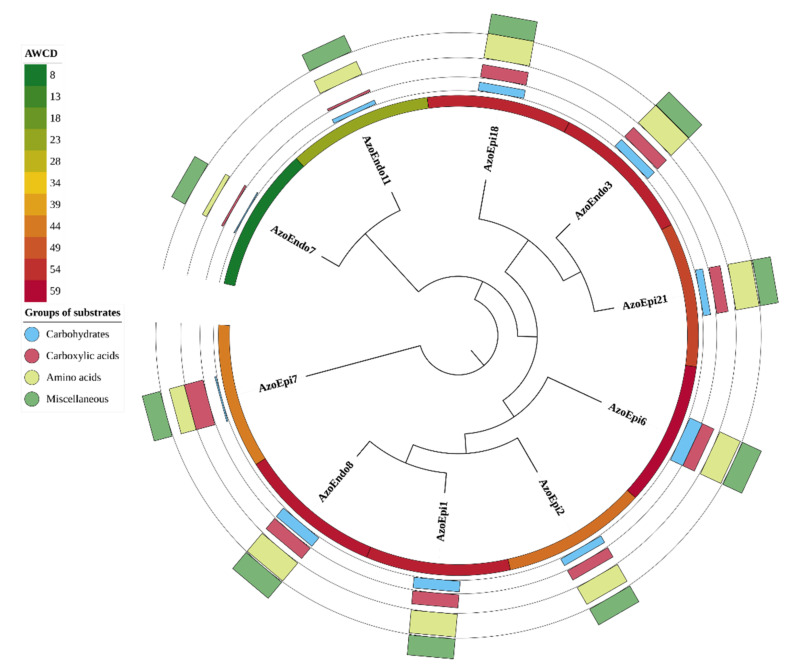
Phenotyping analysis of the studied microorganisms based on the results of GEN III MicroPlates ™ analysis. Clustering analysis tree created on the basis of the results of the use of all substrates after the correction with negative reaction control (UPGMA, Euclidean similarity index). Inner circle (color gradient) presents AWCD—average well color development. Multivalued bar charts present the percent use of different groups of substrates: carbohydrates, carboxylic acid, amino acids, and miscellaneous (each line presents a different group of substrates, as shown in the legend).

**Figure 2:**
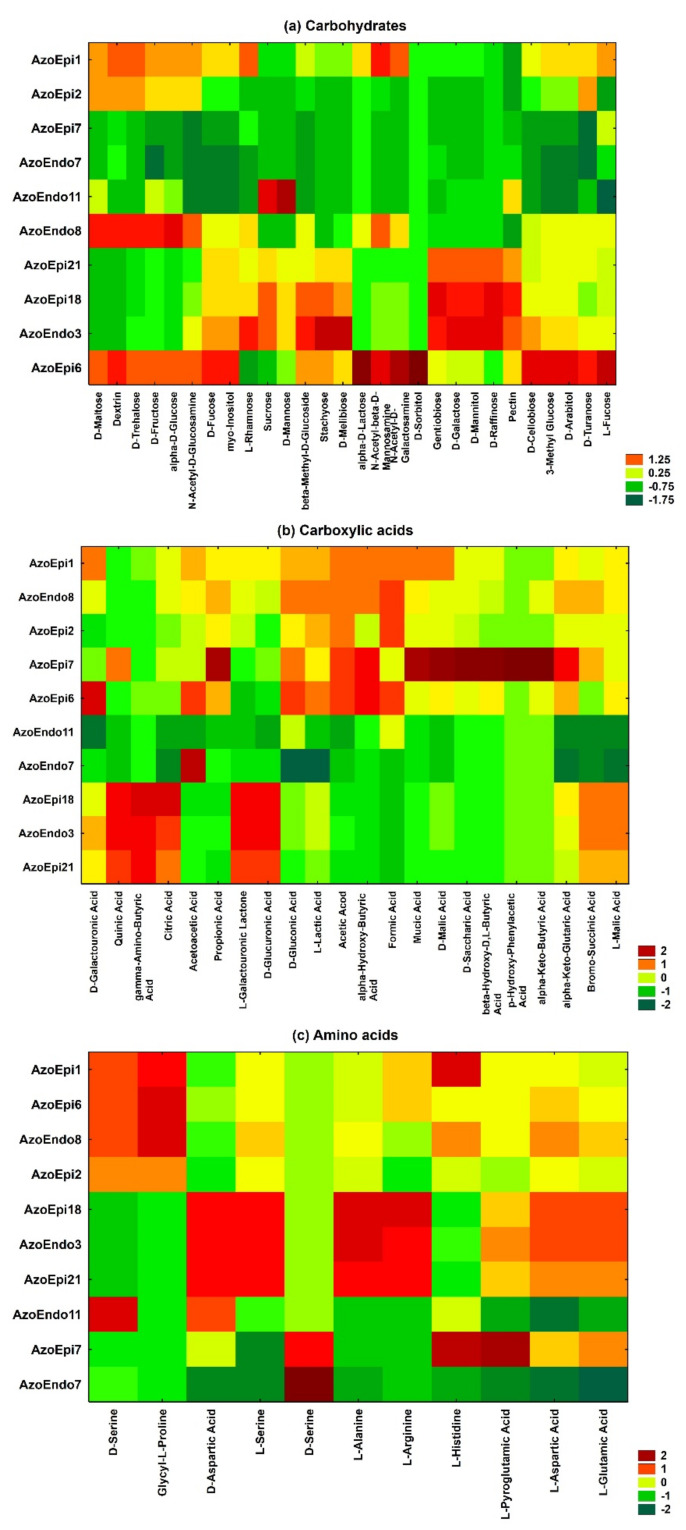
Heatmap of 10 strains showing metabolic profiles for (**a**) carbohydrates, (**b**) carboxylic acid, and (**c**) amino acids after 48 h of incubation. The relative use of selected substrates is depicted by color intensity, based on the legend next to the figure. The highest and lowest consumption rates are indicated by red and green colors, respectively.

The GEN III MicroPlates^TM^ analysis based on average well color density (AWCD) revealed the characteristic metabolic pattern (metabolic fingerprint) of the studied microorganisms during their aerobic metabolism. There were 61 types of carbon sources in the GEN III MicroPlates^TM^, including carbohydrates (28 types), carboxylic acids (22 types), and amino acids (11 types) (Figure 1). The metabolic activity of the analyzed strains demonstrated significant differences in the rates of use of the carbon sources (*p* = 0.0001; Figure 1 and Figure 2). The following order of carbon source use rates was exhibited by the studied bacteria: amino acids > carboxylic acids > carbohydrates. Thus, amino acids were the major carbon sources used by the 10 bacterial strains, while carboxylic acids and carbohydrates were definitely used less efficiently. The statistical analysis showed significant differences in carbohydrate metabolism between the tested bacterial strains (*p* = 0.000001). However, there were no statistically significant differences in the metabolism of amino acids between the tested strains isolated from *Azolla* tissues (*p* = 0.05662). A detailed analysis of the AWCD variation indicated that the carboxylic acid metabolizing activity of the *Micrococcus* sp. AzoEndo7 and *Staphylococcus* sp. AzoEndo11 strains was definitely lower than in the other strains tested. Noteworthy is the carbon metabolism of the *Delftia* sp. AzoEpi7 strain, which uses carboxylic acids and amino acids much more easily than carbohydrates (Figure 1 and Figure 2).

**Figure 3:**
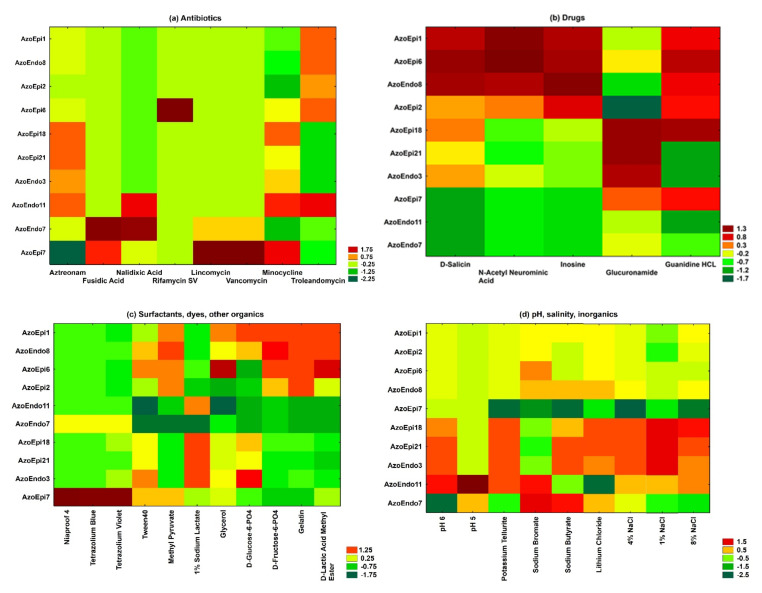
Heatmap of 10 strains showing metabolic profiles for some compounds: (**a**) antibiotics, (**b**) drugs, and (**c**) surfactants, dyes, and other organics, as well as (**d**) pH, salinity, and inorganics after 48 h of incubation. The relative use of selected substrates is depicted by color intensity, based on the legend next to the figure. The highest and lowest consumption rates are indicated by red and green color, respectively.

The phenotypic microarrays identified the differences between the species on the basis of growth at different pH, salinity, assimilation of substrates (for example surfactants, dyes, some toxic ions), and sensitivity to antibiotics. The results obtained revealed that the studied microorganisms varied in their susceptibility to all the antimicrobials. All isolates were 100% susceptible to rifamycin SV and showed low resistance to fusidic acid (Figure 3a). Moreover, all of the isolated bacteria were resistant to aztreonam after incubation for 48 h. *Delftia* sp. AzoEpi7 showed the ability to metabolize the lincomycin and vancomycin antibiotics. Among the bacteria tested, the *Micrococcus* sp. AzoEndo7 and *Staphylococcus* sp. AzoEndo11 showed the lowest ability to metabolize the drugs tested (Figure 3b).

Except for the *Achromobacter* sp. AzoEpi1, *Microbacterium* sp. AzoEpi6, and *Acinetobacter* sp. AzoEndo8, the studied bacteria had the lowest potential to metabolize N-acetylneuraminic acid. The highest metabolic activity at 30 °C for 48 h was demonstrated by the tested strains (except for *Alcaligenes* sp. AzoEpi21, *Staphylococcus* sp. AzoEndo11, and *Bacillus* sp. AzoEndo3) in the case of guanidine HCl. Furthermore, *Achromobacter* sp. AzoEpi1, *Bacillus* sp. AzoEpi2, *Microbacterium* sp. AzoEpi6, *Agrobacterium* sp. AzoEpi18, and *Acinetobacter* sp. AzoEndo8 exhibited metabolic activity against all tested compounds.

The metabolic activity of the strains against the tested surfactants and dyes was low. Among the studied bacteria, only *Micrococcus* sp. AzoEndo7 and *Delftia* sp. AzoEpi7 showed metabolic activity against Niaproof 4. In contrast, Tween40 was metabolized by most bacteria with different degrees of intensity, except for the *Staphylococcus* sp. AzoEndo11. *Achromobacter* sp. AzoEpi1, *Bacillus* sp. AzoEpi2, and *Microbacterium* sp. AzoEpi6 exhibited no ability to metabolize the tested dyes during growth in aerobic conditions at a temperature of 30 °C (Figure 3c).

The *Bacillus* sp. AzoEndo3, *Micrococcus* sp. AzoEndo7, *Staphylococcus* sp. AzoEndo11, *Delftia* sp. AzoEpi7, *Agrobacterium* sp. AzoEpi18, and *Agrobacterium* sp. AzoEpi21 displayed the lowest potential to use the tested organic compounds. The GEN III MicroPlates^TM^ analysis showed that 1% sodium lactate was most actively metabolized by the tested strains. The phenotypic microarrays revealed the lowest potential of the bacteria tested to metabolize D-Glucose-6-PO_4_ (Figure 3c). The GEN III MicroPlates^TM^ analysis evidenced that all the tested bacteria were able to grow at pH 6. In turn, the endophytes *Staphylococcus* sp. AzoEndo11 and *Micrococcus* sp. AzoEndo7 were able to grow at pH 5 (Figure 3d). The analysis of the entire salinity set on the GEN III MicroPlates^TM^ (1–8% NaCl) demonstrated a clear pattern of preference of consumption of multiple sodium chloride solutions in aerobic conditions by the tested bacteria. A slight decrease in growth was noted at the concentration of 4% and 8% NaCl for the *Delftia* sp. AzoEpi7 (Figure 3d).

The phenotypic microarrays identified the differences in the profiles between the species in terms of growth in the presence of some ions: lithium chloride, potassium tellurite, sodium butyrate, and bromate. The GEN III MicroPlates^TM^ analysis showed low activity of the *Delftia* sp. AzoEpi7 to metabolize all these inorganic compounds in aerobic conditions. In turn, high metabolic activity in aerobic conditions was detected in the *Micrococcus* sp. AzoEndo7 (Figure 3d).

**Figure 4:**
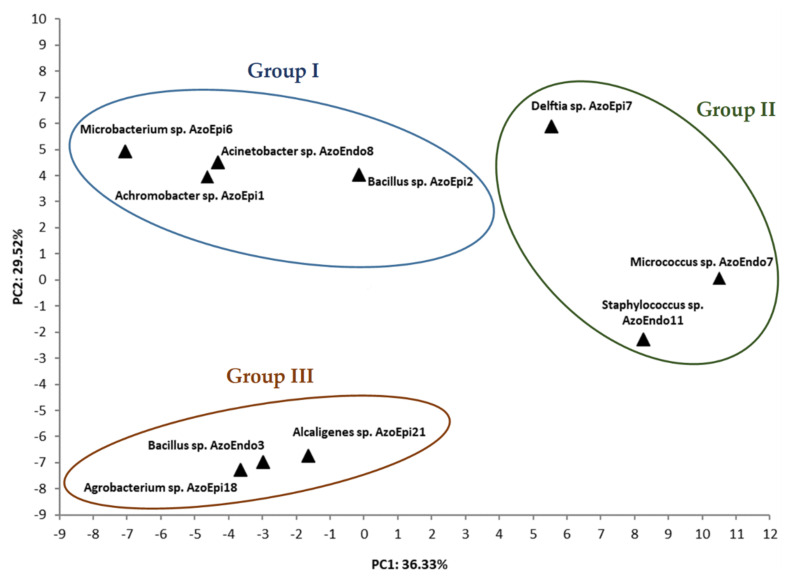
Principal components analysis (PCA) of the Biolog GEN III system for the studied strains after 48h of incubation at 30 °C on GEN III MicroPlates ™; Principal Component 1 (PC1) plotted against Principal Component 2 (PC2) generated by PCA shows the different patterns of 71 carbon sources and 23 susceptibility factors in the studied microorganisms.

The principal component analysis (PCA) evidenced that PC1 and PC2 extracted from the 94 factors (71 carbon sources and 23 susceptibility factors present on the GenIII MicroPlates^TM^) explain 36.33% and 29.52% of the variable variance, respectively, with a rather high cumulative variance contribution rate of 65.85. PCA revealed distinct differences in the metabolic use of the studied compounds between the strains isolated from *Azolla* sp. On this basis, three groups with a different metabolic profile were distinguished, which reflected different structures and characteristics of metabolic activity. As shown in Figure 4, the first group formed the following strains: *Microbacterium* sp. AzoEpi6, *Acinetobacter* sp. AzoEndo8, *Achromobacter* sp. AzoEpi1, *Bacillus* sp. AzoEpi2, and *Delftia* sp. AzoEpi7 with a positive correlation. The second group of strains, *Bacillus* sp. AzoEndo3, *Alcaligenes* sp. AzoEpi21, and *Agrobacterium* sp. AzoEpi18, were located on the negative side of PC2, and the first and third group of strains (*Micrococcus* sp. AzoEndo7 and *Staphylococcus* sp. AzoEndo11) were located on the different sides of PC1. Noteworthy, the *Delftia* sp. AzoEpi7 strain was characterized by a different metabolic profile than the metabolic profiles of the strains divided into three groups. These strains were located on the positive side of PC1 and PC2 as the four groups with the specific metabolisms profile.

### 3.2. Tolerance of Microorganisms to Heavy Metals

The studied strains exhibited various levels of tolerance to the selected metals (Table 2). *Agrobacterium* sp. AzoEpi18 appeared to be the most resistant isolate growing unaffected at all concentrations of the metals with the exception of the highest dose of Pb. Similarly, *Achromobacter* sp. AzoEpi1 showed the highest resistance (all doses of 5 metals) except for 10 mg Au L^−1^. The next level of resistance (4 out of 6 metals) was exhibited by *Microbacterium* sp. AzoEpi6 (affected by Ni and Au), *Alcaligenes* sp. AzoEpi21 (reduced growth at 200 mg L^−1^ Pb and Au), *Acinetobacter* sp. AzoEndo8 (lower growth at Cd and Au), and *Delftia* sp. AzoEpi7 (growth only at 0.5 mg L^−1^ Cd and up to 200 mg L^−1^ Pb). The other isolates were influenced by Pb, Cd, Ni, and Au—both *Bacillus* sp. isolates tolerated the full ranges of Cr and Ag doses, while *Staphylococcus* sp. AzoEndo11 and *Micrococcus* sp. AzoEndo7 were the most sensitive. The former grew in the whole range of Cd and Ag, while the latter grew only in the full range of the Ag doses. It can be concluded that all the bacteria grew well in the Ag treatment, and eight strains, the exceptions being *Staphylococcus* sp. AzoEndo11 and *Micrococcus* sp. AzoEndo7, grew efficiently in the Cr (VI) treatment.

Despite the lower tolerance of some isolates, we decided to determine their enzymatic activities and IAA production in the full range of metal doses to check their metabolism in very unfavorable conditions.

### 3.3. Synthesis of IAA under Heavy Metal Stress

**Figure 5:**
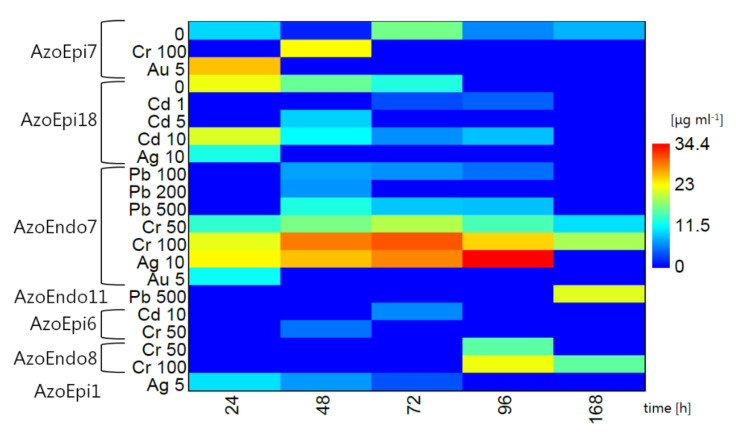
IAA production by the studied isolates under various treatments over time.

Among the isolates, only *Delftia* sp. AzoEpi7 and *Agrobacterium* sp. AzoEpi18 were able to produce IAA in natural conditions. Initially, *Delftia* sp. synthesized 9.71 μg mL^−1^ versus 22.26 μg mL^−1^ of IAA produced by *Agrobacterium* sp. With time, these levels declined in the latter strain to 12.89 μg mL^−1^ and we did not record any phytohormone after 96 h at the end of the experiment. In the case of *Delftia* sp., after a strong decrease to 1.46 μg mL^−1^, this isolate started to produce more IAA up to 16.69 μg mL^−1^ at 77 h followed by a decline to 8.12 μg mL^−1^ at the end of experiment (Figure 5).

The response of the isolates to heavy metals was reflected in various levels of IAA production; some strains released IAA, even though they did not produce this phytohormone in the control treatment. In the presence of Ni, no isolates were able to produce IAA.

Upon exposure to Pb, only *Micrococcus* sp. AzoEndo7 and *Staphylococcus* sp. AzoEndo11 showed IAA production. The former strain showed a similar pattern at 100 and 500 mg Pb L^−1^—the IAA production started at 7.24 and 12.80 μg mL^−1^ after 48 h and decreased for the next 48 h to 4.98 and 8.66 μg mL^−1^, respectively. After the exposure to 200 mg Pb L^−1^, these microorganisms produced IAA only after 48 h (6.65 μg mL^−1^). In the case of *Staphylococcus* sp., it took 168 h to produce IAA, but a high IAA level of 21.55 μg mL^−1^ was achieved (Figure 5). Since 1000 mg Pb L^−1^ strongly affected most strains (Table 1), we did not record any IAA evolution in this treatment.

In the case of the Cd treatment, only *Agrobacterium* sp. AzoEpi18 and *Microbacterium* sp. AzoEpi6 were able to produce IAA. No IAA was detected at the lowest dose of Cd. *Microbacterium* sp. synthesized 6.19 μg mL^−1^ of the phytohormone only after 72 h at the highest dose of Cd. IAA production by *Agrobacterium* sp. was recorded in the presence of 1–10 mg Cd L^−1^ and was linearly related to the metal dose. The lowest levels of IAA (up to 4.35 μg mL^−1^) were recorded at 1 mg Cd L^−1^ followed by 9.46 μg mL^−1^ (5 mg Cd L^−1^), and the highest and longest observed (up to 96 h) yield was noted at 10 mg Cd L^−1^ (on average 11.97 μg mL^−1^). The latter treatment showed initially a high IAA concentration of 21.34 μg mL^−1^, which further decreased to 8.70 μg mL^−1^ (Figure 5).

During the exposure to Cr (VI) ions, four isolates were able to synthesize IAA, namely *Acinetobacter* sp. AzoEndo8, *Micrococcus* sp. AzoEndo7 (both in the whole range of the metal dose), *Delftia* sp. AzoEpi7 (100 mg Cr L^−1^), and *Microbacterium* sp. AzoEpi6 (50 mg Cr L^−1^). In the case of *Acinetobacter* sp., it took 96 h to initiate the production of the phytohormone—15.65 μg mL^−1^ (lower dose) and up to 22.38 μg mL^−1^ (higher dose). *Micrococcus* sp. was very efficient in the release of IAA, as we observed production of the phytohormone throughout the experiment (168 h in total). At 50 mg Cr L^−1^, the concentration of the phytohormone was in the range of 10.13–19.54 μg mL^−1^ with a maximum at 72 h. In the case of the higher metal dose, the amount of IAA ranged between 19.12 and 30.59 μg mL^−1^ with a maximum in the middle of the experiment as well. The efficiency of *Delftia* sp. in the synthesis was also high (22.93 μg mL^−1^), but it was recorded only after 48 h at 100 mg Cr L^−1^. The weakest production of IAA was noted in the case of *Microbacterium* sp., where the concentration amounted to 5.15 μg mL^−1^ after 48 h at the onset of the experiment (Figure 5).

Three strains produced the phytohormone after the exposure to Ag. *Achromobacter* sp. AzoEpi1 displayed continuous synthesis of IAA decreasing from 10.21 to 3.68 μg mL^−1^ during 72 h of the experiment at 5 mg Ag L^−1^. In the presence of 10 mg Ag L^−1^, *Agrobacterium* sp. AzoEpi18 released 12.59 μg mL^−1^ of IAA only during the first 24 h of the metal stress. The most efficient was *Micrococcus* sp. AzoEndo7 showing continuous and increasing synthesis of IAA in the range of 23.10–34.44 μg mL^−1^ during 96 h of the experiment (Figure 5).

Au had a negative impact on the studied microbiome; only two strains synthesized IAA at 5 mg Au L^−1^, namely *Delftia* sp. AzoEpi7 (25.77 μg mL^−1^) and *Micrococcus* sp. AzoEndo7 (11.92 μg mL^−1^), both only during the first 24 h of the experiment (Figure 5).

To summarize, this experiment revealed that *Micrococcus* sp. AzoEndo7 was the most efficient strain in IAA synthesis, as it was able to produce the phytohormone at the exposure to three metals (seven treatments in total). The second most efficient strain was *Agrobacterium* sp. AzoEpi18 (control and two metals, five treatments). The lowest performance was recorded for *Staphylococcus* sp. AzoEndo11 and *Achromobacter* sp. AzoEpi1 (one treatment). It is also interesting that *Micrococcus* sp. was very efficient in IAA production at 100 mg Cr L^−1^ even though this dose of metal had a negative impact on its growth (Table 1).

### 3.4. Cellulolytic Activities in the Presence of Heavy Metals

As all non-treated strains exhibited activities of cellulases, xylanases, and proteases, we focused on the analysis of differences between activities (respectively: CMC_diff_, XYL_diff_ and PROT_diff_) for a given metal treatment and appropriate values for the non-treated strain. We present these data as means with minimum and maximum values.

**Figure 6:**
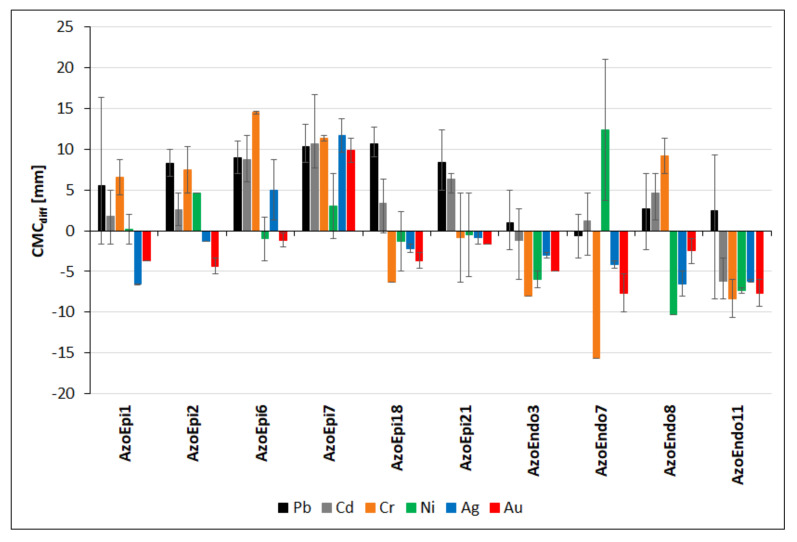
Effect of the metals applied in the specified range of doses (in mg L^−1^) on the cellulolytic activities of the selected strains expressed as differences in the size of halos (in mm) vs. the untreated control. Positive values of bars—stimulation of the activity; negative values of bars—inhibition of the activity. Positive whisker—maximum difference; negative—minimum. See Table 1 for strain codes.

In the case of Pb, all the epiphytes showed significantly higher mean CMC_diffs_ values than the endophytes (*p* < 0.001, Figure 6). In most cases, cellulase activity was stimulated in the presence of Pb; we only recorded slightly smaller halos in some treatments of *Achromobacter* sp. AzoEpi1, *Bacillus* sp. AzoEndo3, *Micrococcus* sp. AzoEndo7, *Acinetobacter* sp. AzoEndo8, and *Staphylococcus* sp. AzoEndo11. *Delftia* sp. AzoEpi7 and *Agrobacterium* sp. AzoEpi18 were the most efficient strains in cellulose degradation, while *Micrococcus* sp. AzoEndo7 was the least efficient (Supplementary Figure S1). In the presence of Cd, 8 strains displayed stimulation of cellulose degradation, compared to the control (CMC_diff_ = 1.17–10.67 mm); only *Bacillus* sp. AzoEndo3 and *Staphylococcus* sp. AzoEndo11 showed smaller decolorization zones resulting in CMC_diff_ = −1.17 and −6.17 mm, respectively (*p* < 0.01, Figure 6). The strongest activities were recorded for *Microbacterium* sp. AzoEpi6 and *Delftia* sp. AzoEpi7 (all doses of Cd), while strong inhibition of cellulase was achieved by *Staphylococcus* sp. AzoEndo11 (all doses of metal, Supplementary Figure S1). The exposure to Cr revealed two equal groups of strains: five with positive CMC_diffs_ (*Achromobacter* sp. AzoEpi1, *Bacillus* sp. AzoEpi2, *Microbacterium* sp. AzoEpi6, *Delftia* sp. AzoEpi7, and *Acinetobacter* sp. AzoEndo8) and five showing decreased cellulase activity (*Agrobacterium* sp. AzoEpi18, *Alcaligenes* sp. AzoEpi21, *Bacillus* sp. AzoEndo3, *Micrococcus* sp. AzoEndo7, and *Staphylococcus* sp. AzoEndo11) (*p* < 0.01, Figure 6). In the case of *Alcaligenes* sp. AzoEpi21, 50 mg Cr L^−1^ led to reduction of cellulose degradation, which was significantly higher at 100 mg Cr L^−1^ (*p* < 0.001). In the case of the other strains, we did not record any significant effect of the metal dose. Again, *Microbacterium* sp. AzoEpi6 and *Delftia* sp. AzoEpi7 were the most efficient strains in cellulose degradation at the exposure to Cr, whereas the most inhibiting response was recorded for *Micrococcus* sp. AzoEndo7 (Supplementary Figure S1). Ni had a negative effect on cellulase activity, as only 4 strains out of 10 (*Achromobacter* sp. AzoEpi1, *Bacillus* sp. AzoEpi2, *Delftia* sp. AzoEpi7, and *Micrococcus* sp. AzoEndo7) exhibited CMC_diffs_ > 0 (*p* < 0.001, Figure 6). *Micrococcus* sp. AzoEndo7 was the most efficient with the highest CMC_diff_ of 21 mm at 50 mg Ni L^−1^, while *Acinetobacter* sp. AzoEndo8 showed the highest inhibition of cellulolytic activity (CMC_diff_ = −10.3 mm, both Ni doses) (Supplementary Figure S1). Both noble metals strongly inhibited the potential of the studied strains for cellulose degradation (Figure 6). In the case of Ag, only *Microbacterium* sp. AzoEpi6 and *Delftia* sp. AzoEpi7 had positive CMC_diffs_ (on average 5 and 11.7 mm, respectively, *p* < 0.01). The strongest inhibition of cellulase was recorded for *Achromobacter* sp. AzoEpi1, *Acinetobacter* sp. AzoEndo8, and *Staphylococcus* sp. AzoEndo11 (CMC_diff_ ranging between −5 and −8 mm) (Supplementary Figure S1). During the exposure to Au, only *Delftia* sp. AzoEpi7 exhibited stimulated cellulolytic activity (CMC_diff_ = 8–11 mm, *p* < 0.001). The strongest inhibition of this activity in relation to the control was noted for *Micrococcus* sp. AzoEndo7 and *Staphylococcus* sp. AzoEndo11 at 10 mg Au L^−1^ (Supplementary Figure S1).

Our analysis of cellulolytic activity allowed us to arrange the isolates from the most resistant, i.e., active under heavy metal stress, to the most sensitive, which represents phenotype shifting towards both resistance and sensitivity. The strongest activity was exhibited by *Delftia* sp. AzoEpi7 showing stimulation of cellulose degradation (CMC_diff_: 7.0–16.7 mm) in all metal treatments (the weakest at Ni), followed by *Microbacterium* sp. AzoEpi6 (five metals, CMC_diff_: 1.3–14.7 mm, a negative effect of one dose of Ni), *Bacillus* sp. AzoEpi2 (four metals, CMC_diff_: 0.7–10.3 mm, reduced activity at Ag and Au), *Alcaligenes* sp. AzoEpi21 (four metals, CMC_diff_: 4.7–12.3 mm, affected at Ag, Au, 50 mg L^−1^ Cr and Ni), *Achromobacter* sp. AzoEpi1 (4 metals, CMC_diff_: 0.7–16.3 mm, additional reduction at Ag, Au, Cd 1, Ni 50, and Pb 1000 mg L^−1^), *Acinetobacter* sp. AzoEndo8 (3 metals, CMC_diff_: 1.3–11.3 mm, low activity at Ag, Au, and Ni, additionally affected at Pb 200 mg L^−1^), *Agrobacterium* sp. AzoEpi18 (two metals—Cd and Pb, CMC_diff_: 4–12.7 mm, affected at Cd 10 mg L^−1^), *Micrococcus* sp. AzoEndo7 (two metals—Cd and Ni, CMC_diff_: 1.3–21 mm, strong reduction at Cr, CMC_diff_: −15.7 mm), *Bacillus* sp. AzoEndo3 (active at Pb 200–500 mg L^−1^, CMC_diff_ up to 5 mm, Cd 5–10 mg L^−1^, CMC_diff_ up to 2.7 mm), and *Staphylococcus* sp. AzoEndo11 (active only at Pb 200–1000 mg L^−1^, CMC_diff_ up to 8.7 mm).

**Figure 7:**
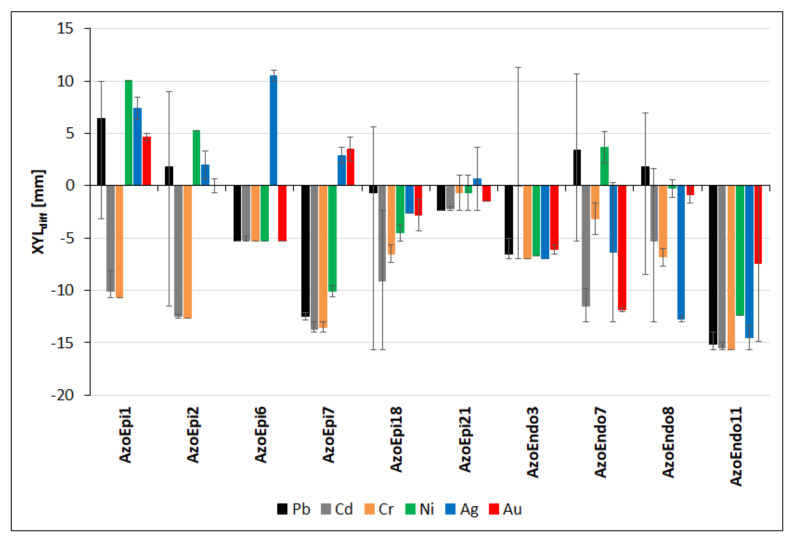
Effect of the metals applied in the specified range of doses (in mg L^−1^) on the xylanolytic activities of the selected strains expressed as differences in the size of halos (in mm) vs. the untreated control. Positive values of bars—stimulation of the activity; negative values of bars—inhibition of the activity. Positive whisker—maximum difference; negative—minimum. See Table 1 for strain codes.

The studied isolates displayed various phenotypes in terms of their potential for xylan degradation. In the presence of Pb, 4 out of the 10 strains exhibited higher decomposition of xylan in relation to the control (*p* < 0.01). The highest average XYL_diff_ was recorded for *Achromobacter* sp. AzoEpi1 (6.4 mm), followed by *Micrococcus* sp. AzoEndo7 (3.4 mm), *Bacillus* sp. AzoEpi2, and *Acinetobacter* sp. AzoEndo8 (both 1.8 mm). However, reduced decomposition rates were noted in the 1000 mg Pb L^−1^ treatments (Supplementary Figure S2). The strongest inhibition of xylanase activity was noted for *Delftia* sp. AzoEpi7 (−12.5 mm) and *Staphylococcus* sp. AzoEndo11 (−15.1 mm) at all metal doses (Figure 7, Supplementary Figure S2). The exposure to Cd had a strong negative effect on xylan degradation in all strains (Figure 7). *Achromobacter* sp. AzoEpi1, *Bacillus* sp. AzoEpi2, *Delftia* sp. AzoEpi7, *Micrococcus* sp. AzoEndo7, and *Staphylococcus* sp. AzoEndo11 showed the strongest enzymatic inhibition (XYL_diff_ < −10 mm (*p* < 0.01, Figure 7). *Bacillus* sp. AzoEndo3 was the least affected, as it exhibited stimulation at 0.5 (XYL_diff_: 2 mm) and 1 mg Cd L^−1^ (XYL_diff_: 11.3 mm) (*p* < 0.001, Supplementary Figure S2). Cr also negatively influenced the xylanolytic ability of the studied strains. The most sensitive strains were *Achromobacter* sp. AzoEpi1, *Bacillus* sp. AzoEpi2, *Delftia* sp. AzoEpi7, and *Staphylococcus* sp. AzoEndo11 exhibiting XYL_diff_ < −10 mm (*p* < 0.001, Figure 7). *Alcaligenes* sp. AzoEpi21, in turn, was the only strain displaying increased xylan degradation ability at the exposure to 50 mg Cr L^−1^ (*p* < 0.001, Supplementary Figure S2). In the presence of Ni, only 3 out of the 10 strains exhibited positive average values of XYL_diff_—*Achromobacter* sp. AzoEpi1 (10 mm), *Bacillus* sp. AzoEpi2 (5.3 mm), and *Micrococcus* sp. AzoEndo7 (3.7 mm). There were two strains showing the highest inhibition of xylan degradation, namely *Delftia* sp. AzoEpi7 (−10 mm) and *Staphylococcus* sp. AzoEndo11 (−12.4 mm) (*p* < 0.001, Figure 7). There was no significant dose effect on the observed strains responses (*p* = 0.11, Supplementary Figure S2). The studied strains showed various responses to Ag. Five of them, namely *Achromobacter* sp. AzoEpi1, *Bacillus* sp. AzoEpi2, *Microbacterium* sp. AzoEpi6, *Delftia* sp. AzoEpi7, and *Alcaligenes* sp. AzoEpi21, were able to decompose xylan more efficiently than the non-treated strains (*p* < 0.01). *Microbacterium* sp. AzoEpi6 (XYL_diff_: 10.5 mm) was the most efficient strain, while *Acinetobacter* sp. AzoEndo8 and *Staphylococcus* sp. AzoEndo11 showed much lower potential XYL_diff_s < −10 mm (*p* < 0.01, Figure 7). *Alcaligenes* sp. AzoEpi21 and *Micrococcus* sp. AzoEndo7 showed negative differences at 5 mg Ag L^−1^ (*p* < 0.001, Supplementary Figure S2). During the exposure to Au, only *Achromobacter* sp. AzoEpi1 and *Delftia* sp. AzoEpi7 exhibited positive values of XYL_diff_—4.7 and 3.5 mm, respectively (*p* < 0.001, Figure 7). In turn, the highest negative value was recorded for *Micrococcus* sp. AzoEndo7 (XYL_diff_: −11.8 mm). No effect of 5 mg Au L^−1^ was demonstrated in the case of *Acinetobacter* sp. AzoEndo8 and *Staphylococcus* sp. AzoEndo11, while an average difference of xylanase activity of 0 was recorded for *Bacillus* sp. AzoEpi2 (Supplementary Figure S2).

In summary, the tested metals had strong and negative effects on the xylanolytic activity of the selected strains allowing us to categorize them and identify resistant and sensitive phenotypes. *Achromobacter* sp. AzoEpi1 was the most resistant strain, as it was able to produce more xylanase in the presence of four metals (XYL_diff_ = 4.3–11.3 mm, activity inhibited only in the Cd treatment). It was followed by *Bacillus* sp. AzoEpi2 (four metals, XYL_diff_ = 0.7–9 mm, affected by Cd, Cr, and 10 mg Au and 1000 mg Pb L^−1^), *Micrococcus* sp. AzoEndo7 (2 metals—Pb, Ni; XYL_diff_ = 4.8–10.7 mm, affected by 100 mg Ni and 1000 mg Pb L^−1^), *Acinetobacter* sp. AzoEndo8 (three doses of Pb and one dose of Cd and Ni, XYL_diff_ = 1.7–7 mm), *Delftia* sp. AzoEpi7 (two metals—Ag, Au; XYL_diff_ = 2.2–4.7 mm), and *Microbacterium* sp. AzoEpi6 (one metal: Ag; XYL_diff_ = 10–11 mm). *Alcaligenes* sp. AzoEpi21, *Agrobacterium* sp. AzoEpi18, and *Bacillus* sp. AzoEndo3 showed some activities in single treatments (XYL_diff_ = 1, 2.33–5.67, and 2.3–11.3 mm, respectively), while *Staphylococcus* sp. AzoEndo11 displayed reduction of xylanolytic activity in all treatments.

### 3.5. Proteolytic Activities in the Presence of Heavy Metals

**Figure 8:**
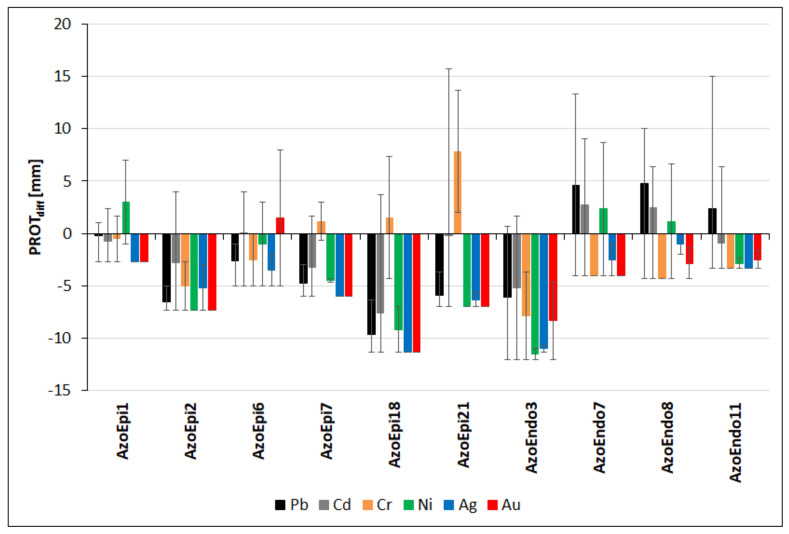
Effect of the metals applied in the specified range of doses (in mg L^−1^) on the proteolytic activities of the selected strains expressed as differences in the size of halos (in mm) vs. the untreated control. Positive values of bars—stimulation of the activity; negative values of bars—inhibition of the activity. Positive whisker—maximum difference; negative—minimum. See Table 1 for strain codes.

The heavy metals had a significant effect on the potential of the studied strains for use of proteins, resulting in various phenotypes. The exposure to Pb led to stimulation of proteolytic activities in relation to the control in the case of 3 endophytic strains: *Micrococcus* sp. AzoEndo7, *Acinetobacter* sp. AzoEndo8, and *Staphylococcus* sp. AzoEndo11 (PROT_diff_: 4.6, 4.8, and 2.3 mm, respectively). In these strains, significant inhibition of protease activity was observed at 1000 mg Pb L^−1^ (*p* < 0.01, Supplementary Figure S3). The other strains exhibited lower proteolytic activities in comparison to the control (*p* < 0.001). *Achromobacter* sp. AzoEpi1 exhibited slightly higher activities than in the control (PROT_diff_ = 0.2–1.0 mm) and negative (−2.7 mm) at 1000 mg Pb L^−1^ (Supplementary Figure S3). The lowest difference of −9.7 mm was recorded for *Agrobacterium* sp. AzoEpi18 (Figure 8). In the case of Cd, 3 out of the 10 strains showed positive values of PROTdiff, i.e., *Micrococcus* sp. AzoEndo7 and *Acinetobacter* sp. AzoEndo8 exhibited differences of 2.5–2.8 mm (*p* > 0.05) and *Microbacterium* sp. AzoEpi6—0.08 mm. The strongest inhibition of proteases was caused by *Agrobacterium* sp. AzoEpi18 (PROT_diff_ = -7.6 mm, *p* < 0.001, Figure 8). The lowest differences were recorded at 10 mg Cd L^−1^ in the case of all strains (Supplementary Figure S3). During the exposure to Cr, three strains exhibited increased proteolytic activities. The highest positive PROT_diff_ of 7.8 mm was recorded for *Alcaligenes* sp. AzoEpi21, while the most negative value of −7.8 mm was noted for *Bacillus* sp. AzoEndo3 (*p* < 0.01, Figure 8). In the case of *Microbacterium* sp. AzoEpi6 and *Delftia* sp. AzoEpi7, negative and positive differences were noted at 100 mg Cr L^−1^ and 50 mg Cr L^−1^, respectively (*p* < 0.01, Supplementary Figure S3). The exposure to Ni led to identification of three strains with stimulated proteolytic activity to maximum PROT_diff_ of 3.0 mm in the case of *Achromobacter* sp. AzoEpi1. However, negative differences were observed for all strains at 100 mg Ni L^−1^ (*p* < 0.001, Supplementary Figure S3). *Bacillus* sp. AzoEndo3 exhibited the strongest inhibition of protease (PROT_diff_: −11.5 mm, *p* < 0.001, Figure 8). The noble metals had strong inhibiting effect on proteolytic activities; in the case of Ag, all strains exhibited varying negative differences (*p* < 0.01, Figure 8). *Acinetobacter* sp. AzoEndo8 was the most resistant (PROT_diff_: −1 mm) and *Agrobacterium* sp. AzoEpi18 was the most sensitive (PROT_diff_: −11.3 mm). There were no significant effects of the Ag dose on the recorded differences (*p* = 0.146, Supplementary Figure S3). In the case of Au, only *Microbacterium* sp. AzoEpi6 displayed increased enzymatic activity (PROT_diff_: 1.5 mm). The other strains exhibited significantly lower values ranging from −2.5 to −11.3 mm (*p* < 0.001, Figure 8). In most cases, the inhibition was dose-related (*p* < 0.001, Supplementary Figure S3).

To summarize, we found that all metals had very strong negative effects on the proteolytic activity. It was inhibited by Ag and Au in all strains with the exception of *Microbacterium* sp. AzoEpi6 (Au treatment). In addition, the analysis of the calculated differences allowed us to classify the strains according to their metabolic sensitivity (protease) to the studied metals, which allowed identification of various phenotype shifts. Based on the differences, we found the highest resistance of *Micrococcus* sp. AzoEndo7 and *Acinetobacter* sp. AzoEndo8 showing proteolytic activity in treatments with three metals (PROT_diff_ = 2.7–13.3 and 3.7–10 mm, respectively). *Microbacterium* sp. AzoEpi6 was the third most resistant strain, as it was the only one to show increased activity at 5 mg Au L^−1^ and in single treatments with Cd and Ni (PROT_diff_ = 3–8 mm). *Alcaligenes* sp. AzoEpi21 and *Agrobacterium* sp. AzoEpi18 showed activities in both Cr treatments and at the single Cd dose (PROT_diff_ = 2–15.7 and 3.7–7.3 mm, respectively). They were followed by *Delftia* sp. AzoEpi7 (PROT_diff_ = 1.7–3 mm), *Achromobacter* sp. AzoEpi1 (PROT_diff_ = 0.3–7 mm, low potential in treatments with three metals), and *Staphylococcus* sp. AzoEndo11 (PROT_diff_ = 1–15 mm, one metal). Both *Bacillus* sp. strains showed stimulation of proteolytic activities only at 0.5 and 5 mg Cd L^−1^ (PROT_diff_ = 1.7–4 mm).

## 4. Discussion

Determination of microbial responses to HM stress in terms of metabolism switching is important for the elucidation of the possible role of microorganisms in supporting plants in unfavorable conditions. This study made it possible to screen the phenotype switching possibilities of the analyzed bacteria as metabolic traits, which can help to elucidate their niche adaptation [4]. Although there have been many such studies, not all microbes have been tested yet under exposure to Pb, Cd, Cr, Ni, Ag, and Au. Most studies have only focused on recognition of the PGP potential of newly isolated microbes [11,12,13,14,15,16]. We aimed to reveal this unexplored field to find new potentially metal-tolerant species with concurrent PGP traits. In the presence of pollutants, microbial activity may be altered, leading to a switch of bacterial metabolic pathways [17], probably due to the enormous plasticity of the bacterial genome.

In our study, all strains exhibited the activities of the analyzed enzymes. However, metal stress caused changes in their effectiveness, i.e., phenotype switching. Heavy metals are key drivers of microbial response in terms of phytohormone synthesis and the potential for decomposition of the main constituents of pathogen cells (Cell Wall Degrading Enzymes, CWDE) [6,17,18]. Since metals are often parts of enzymes, their presence may increase enzymatic activity, stimulating plant growth. However, elevated levels of HMs may negatively affect metabolic pathways, lowering metabolism and causing adverse effects to organisms [7,18]. IAA is known to be a regulator that stimulates cell elongation and division, leading to enhanced plant growth. Several factors are involved in auxin metabolism under HM stress: PIN1 protein (auxin efflux carrier), ethylene (stimulation of IAA transport), nitrogen (II) oxide (transport repressor), antioxidant enzymes (activated at HM stress by an auxin conjugate), and hemicellulose (auxins enhance the content of hemicelluloses, which bind metals) [18]. This demonstrates how the microbial phenotype may change in terms of auxin synthesis in the presence of HMs. When a plant is exposed to heavy metals, its auxin level decreases; however, endosymbiotic bacteria can re-supply it and promote plant growth in unfavorable conditions [18]. Cellulose and hemicellulose are important fractions of biomass, being the first and second largest components of lignocellulosic biomass, with a ratio of 40.6–51.2% and 28.5–37.2%, respectively. Efficient degradation of this complex material requires the synergistic action of a group of biocatalysts, including cellulase and endoxylanase [19]. For this reason, it is important to study both enzymes to see microbial efficiency in cell wall decomposition. Many bacteria and fungi produce hydrolytic enzymes targeting organic polymers present in soil and water as well as being part of living organisms. Hence, the enzymatic action of microorganisms allows the elimination of pathogenic organisms by damaging their cell walls and participating in carbon and nitrogen cycling.

In our study, we aimed to reveal the ability of the *A. filiculoides* microbiome to mitigate pollutants (organics and HMs) and its PGP potential. For this purpose, we combined phenotyping with PGP tests to detect changes in bacterial phenotypes in stress conditions. Microorganisms showing high plasticity are highly valuable in bioremediation of polluted sites and enhancement of plant production.

The most metal-resistant *Agrobacterium* sp. AzoEpi18 showed reduced synthesis of IAA in relation to the control conditions in the Cd treatment and moderate IAA production in the Ag treatment. This strain showed the highest synthesis of auxin (12.89–22.26 μg mL^−1^), which is similar to the values presented in a study conducted by Woźniak and co-authors [20], i.e., up to 22.51 μg mL^−1^ produced by endophytes. Mashiguchi and co-workers [21] showed that *Agrobacterium tumefaciens* enhanced the biosynthesis of IAA when introduced into tomato plants. Similar effects were presented for *Agrobacterium radiobacter* showing tolerance to Pb, Cd, Zn, and Cu [22]. The capability of *Agrobacterium* sp. AzoEpi18 of cellulose degradation was mostly reduced. Higher activities were recorded only in the presence of all concentrations of Pb, and lower levels were detected at 0.5–5 mg Cd L^−1^. Xylanase activity was strongly inhibited by the analyzed metals except for 100–500 mg Pb L^−1^. Increased proteolytic activity of *Agrobacterium* sp. AzoEpi18 was demonstrated in the 5 mg Cd L^−1^ and 50 mg Cr L^−1^ treatments. This strain showed greater efficiency of use of amino acids as a source of carbon and energy than sugars and carboxylic acids. The analysis of the growth conditions of the strain showed that the cells only proliferated at pH 6, but surprisingly efficient growth was identified at all sodium chloride concentrations tested (1–8%). The data of the GEN III MicroPlates^TM^ indicate that *Agrobacterium* sp. AzoEpi18 has metabolic pathways that allow this strain to grow in the presence of lithium chloride, potassium tellurite, sodium bromate, aztreonam, guanidine HCl, and 1% sodium lactate. Unfortunately, this strain cannot be used in an environment contaminated with tetrazolium blue, tetrazolium violet, Niaproof 4 and Tween40 surfactants, as well as drugs and antibiotics, as this strain has not been shown to be able to use these compounds.

The highly metal-resistant *Achromobacter* sp. AzoEpi1 exhibited slight changes in terms of IAA synthesis only at 5 mg Ag L^−1^ in relation to the control. Cellulase activity was stimulated in the presence of four of the tested metals (Pb, Cd, Cr, and Ni). In terms of xylanolytic activity, *Achromobacter* sp. showed the highest stimulation in the presence of three metals in the full range of doses (Ni, Ag, and Au) and at 100–500 mg Pb L^−1^. Xylan degradation was inhibited by Cd and Cr. The potential for protein decomposition was only reduced in the presence of the noble metals and 1000 mg Pb L^−1^, 5 mg Cd L^−1^, and 50 mg Cr and Ni L^−1^. The metabolic patterns of the *Achromobacter* sp. AzoEpi1 strain indicate its similarity to the *Agrobacterium* sp. AzoEpi18, except that the former strain is capable of metabolizing troleandomycin, methyl pyruvate, and gelatin.

*Microbacterium* sp. AzoEpi6 exhibited slight stimulation of IAA release in the Cr (50 mg L^−1^) and Cd (10 mg L^−1^) treatments. Coretto and co-workers [23] demonstrated production of plant growth promoting compounds and tolerance to Zn, Cd, and Pb in *Microbacterium* sp. In addition, the strain had increased cellulase activity in the presence of four metals (all doses of Pb, Cd, Cr, and Ag) and 100 mg Ni L^−1^. However, the xylanolytic activity of this strain was strongly reduced, with stimulation only in the Ag treatments. An increase in proteolytic ability was demonstrated in the case of 1–5 mg Cd L^−1^, 50 mg Ni L^−1^, and 5 mg Au L^−1^. The GEN III MicroPlates^TM^ data for *Microbacterium* sp. AzoEpi6 indicated its high metabolic (phenotypic) similarity to the two strains discussed previously (*Achromobacter* sp. AzoEpi1 and *Agrobacterium* sp. AzoEpi18).

We did not detect any IAA synthesis by *Alcaligenes* sp. AzoEpi21; however, the strain *Alcaligenes* isolated by Fatima and co-workers produced this auxin under salinity stress, with an increase to 33.92 μg mL^−1^ at 700 mM NaCl [16]. All doses of Pb and Cd stimulated cellulose degradation by this strain, while the noble metals and the lower doses of Cr and Ni inhibited this activity. The heavy metals had a negative effect on xylan degradation, with the exception of 100 mg Pb L^−1^ and 50 mg Cr L^−1^. During the exposure to both doses of Cr (VI), *Alcaligenes* sp. exhibited higher proteolytic activity than in the control variant. Similar observations were recorded for 5 mg Cd L^−1^. Our results have indicated that this strain probably prefers amino acids as carbon and energy sources and, to a lesser extent, carboxylic acids and carbohydrates. The *Alcaligenes* sp. AzoEpi21 has shown efficient growth at pH 6 at the concentration of the sodium chloride solutions tested. The Biolog GEN III MicroPlates^TM^ analysis revealed the presence of metabolic pathways allowing removal of aztreonam, glucuronamide, D-Salicin, Tween40, and 1% sodium lactate from the environment.

Cr (VI) contributed to elevated production of IAA by *Acinetobacter* sp. AzoEndo8 in comparison to the control. Its cellulolytic activity was completely reduced in the presence of three metals (Ni, Ag, and Au) and 500 mg Pb L^−1^. The xylanolytic activity of *Acinetobacter* sp. was strongly affected by the tested metals; it was only stimulated by the 100–500 mg Pb L^−1^, 1 mg Cd L^−1^, and 50 mg Ni L^−1^ treatments. The potential of *Acinetobacter* sp. AzoEndo8 for stronger protease degradation was noted for Pb (100–500 mg L^−1^), Cd (0.5–5 mg L^−1^), and 50 mg Ni L^−1^. Wang and co-workers [19] tested the activity of xylanase XynA isolated from *Bacillus* sp. KW1 exposed to different physical and chemical factors. They noted 18% inhibition of the activity in the presence of Ni. This strain, like most of the tested strains, was tolerant to pH and salt concentration. *Acinetobacter* sp. AzoEndo8 is similar to *Achromobacter* sp. AzoEpi1 and *Microbacterium* sp. AzoEpi6 in terms of the ability to metabolize gelatin.

*Delftia* sp. AzoEpi7 was one of the strains showing IAA production in natural conditions (1.46–16.69 μg mL^−1^). Woźniak and co-authors [20] also presented IAA production by various *Delftia* sp. strains ranging between 1.02 and 22.51 μg mL^−1^. In our study, the presence of Au (5 mg L^−1^) and Cr (100 mg L^−1^) had strong effects on IAA production by *Delftia* sp. in comparison to the absence of any metal, but this was only visible for a short time. A study conducted by Morel and co-workers [24] reported IAA production by *Delftia* sp. JD2 only at 4 mg Cr (VI) L^−1^, while no auxin was detected at 100 mg Cr L^−1^. *Delftia* sp. AzoEpi7 exhibited stimulation of cellulolytic activity in the case of 5 metals (all doses) and at 100 mg Ni L^−1^, which makes it the best-adapted strain in our study. The xylanolytic activity of AzoEpi7 was strongly reduced and only the presence of the noble metals stimulated it above the levels in the control. In the case of *Delftia* sp., increased protease activity was noted during the exposure to 1 mg Cd L^−1^ and 50 mg Cr L^−1^.

This strain has a very interesting phenotype profile and expression thereof. On the one hand, *Delftia* sp. AzoEpi7 tolerates a smaller range of pH factors or sodium chloride concentrations; on the other hand, it has the ability to degrade essential antibiotics: lincomycin, vancomycin, and minocycline as well as other substances: Niaproof 4, tetrazolium blue, and tetrazolium blue.

A positive effect of IAA on plant growth was demonstrated in *Sedum alfredi* Hance in the presence of endophytic *Bacillus fluorescens* Sasm5. Moreover, this bacterium possesses metal transporters: ZRT/IRT-like protein (ZIP) and heavy metal ATPase (HMA) participating in Cd uptake and translocation, which indicates that the bacterium directly regulates the expression of putative key Cd uptake and transport genes to enhance Cd accumulation in plants [25]. Fatima and Ahmed [26] demonstrated the potential of *Sporosarcina saromensis* and two strains of *Bacillus cereus* to stimulate the growth of *Helianthus annuus* L. exposed to Cr (VI) by production of auxin. He and co-workers [27] suggested that two strains of *Bacillus* sp., QX8 and QX13, exhibited strong resistance to Cd and Pb and produced IAA. In our study, however, neither strain of *Bacillus* sp. showed any IAA synthesis, but both grew well in the presence of these metals. Similar findings were presented by Yu and co-authors [28] and Efe [29], where not all *Bacillus* strains produced this auxin. The effect of Au on IAA was studied by Panichikkal and co-workers [30]. In their study, gold nanoparticles (AuNPs) synthesized by *Bacillus subtilis* SJ15 were introduced into the growing medium of *Pseudomonas monteilii* and *Vigna unguiculata*. The study showed increased production of IAA by the bacterium at 50 μg mL^−1^ AuNPs and enhanced the growth of the plant. Kumar and co-workers [31] conducted a similar study using silver NPs and *Bacillus cereus* LPR2 and showed positive effects of both factors on maize growth. The cellulolytic activity of *Bacillus* sp. AzoEndo3 was stimulated only at 200–500 mg Pb L^−1^ and 5–10 mg Cd L^−1^, while Cr, Ni, Ag, and Au had inhibiting effects. The epiphytic *Bacillus* sp. exhibited increased cellulose degradation at the exposure to Pb, Cd, Cr, and Ni and inhibition thereof by the noble metals. The potential for xylan degradation in *Bacillus* sp. AzoEpi2 was higher in the presence of Ni, Ag, 100–500 mg Pb L^−1^, and 5 mg Au L^−1^. The exposure to Cd and Cr inhibited this activity. In the case of the endophytic *Bacillus* sp., only 0.5–1 mg Cd L^−1^ had a positive effect on the potential for xylan degradation, whereas the other treatments had an inhibiting effect. Strong inhibition of xylanolytic activity of *Bacillus* GESF-1 in the presence of Hg^2+^, Fe^3+^, Cu^2+^, Cd^2+^, and Zn^2+^ was demonstrated by Menon et al. [12]. The proteolytic activity of this strain was higher in the control only at 0.5 and 5 mg Cd L^−1^. The epiphytic *Bacillus* AzoEpi2 displayed the lowest proteolytic activity, as the positive effect of the presence of metals was only observed at 5 mg Cd L^−1^. A study carried out by Sumardi and co-workers [32] demonstrated the inhibiting effects of Ca, Cu, and Fe on proteolytic activity. In contrast, the activity was strongly stimulated in the presence of Mn (II); moreover, additional application of magnetic field with the addition of FeCl_3_ had a positive effect.

*Staphylococcus* sp. AzoEndo11 produced 21.55 μg mL^−1^ IAA in the Pb treatment after the prolonged exposure but not in the control. However, this microbe was proved to produce auxin at a higher level of 38.8 μg mL^−1^ [13]. In addition, its cellulase activity was inhibited most strongly (five metals), as it was higher only at 200–1000 mg Pb L^−1^ in comparison to the control. This was the only strain exhibiting the complete reduction of xylan degradation in all metal treatments. In the case of proteolytic activity, a positive effect on the amount of decomposed protein was only recorded in the 200–500 mg Pb L^−1^ and 0.5 mg Cd L^−1^ treatments.

The Biolog GEN III MicroPlates^TM^ analysis of this strain is extremely interesting. This is the only strain that can grow at pH 5, and also in the tested sodium chloride range (1–8%). This strain cannot grow in the presence of lithium chloride. Among the possible environmental pollutants, this strain only shows the ability to metabolize aztreonam, troleandomycin, nalidixic acid, and 1% sodium lactate.

*Micrococcus* sp. AzoEndo7 was the most sensitive to the tested metals; however, this strain showed the strongest switch in terms of IAA synthesis. It produced no IAA under control conditions. Nevertheless, the exposure to Ag (10 mg L^−1^), Cr (both doses), Pb (100–500 mg L^−1^), and 5 mg Au L^−1^ induced strong/intermediate production of the phytohormone. High production of IAA equal to 154.3 μg mL^−1^ by *Micrococcus aloeverae* DCB-20 was demonstrated by Ahamd and co-workers [33], who indicated the high potential of this microorganism to promote plant growth. The ability of *Micrococcus* sp. AzoEndo7 to degrade cellulose was reduced by Cr, Ag, Au (all doses), and 1 mg Cd L^−1^ but enhanced by 500 mg Pb L^−1^, both doses of Ni, and three doses of Cd. The xylanolytic activity of *Micrococcus* sp. AzoEndo7 was strongly affected by the tested metals; it was only stimulated in the 100–500 mg Pb L^−1^ and 50 mg Ni L^−1^ treatments. Higher potential for protease degradation was recorded for Pb (100–500 mg L^−1^), Cd (0.5–5 mg L^−1^), and 50 mg Ni L^−1^. This strain had the lowest degree of use of carbohydrates, amino acids, and carboxylic acids. Interestingly, it exhibited extremely low growth at pH 5 and 6, which makes it necessary to extend the research to test bacterial growth at different pH. *Micrococcus* sp. AzoEndo7 turned out to be tolerant to the inorganic compounds used. Unfortunately, this strain showed no or extremely low tolerance to drugs, surfactants, organic compounds, and dyes.

Mendoza-Hernández et al. [34] demonstrated that the initial production of IAA by strains isolated from the plant rhizosphere was low (up to 11.5 μg mL^−1^) and strongly affected by the presence of heavy metals. They noted strong stimulation of IAA synthesis in the presence of Cu, Pb, and As, while exposure to Ni, Cr, Cd, and Mn had inhibiting effects. Besides heavy metals, we tested the effects of Ag and Au. An earlier study showed that silver nitrate (V) may both block ethylene, which inhibits the synthesis of IAA, and promote auxin efflux from roots resulting in low levels of the phytohormone [35]. Haddad and co-workers [36] demonstrated inhibited cellulase activity in clay and sandy soil contaminated with Cd, Co, Ni, and Pb. Kandler and co-workers [37] found that soil microbial activity, including cellulases, xylanases, and proteases, was slightly affected by Zn, Cu, Ni, V, and Cd in comparison to enzymes involved in N, P, and S cycling.

During the exposure to Pb, all the epiphytes increased their potential to degrade cellulose, while the endophytes showed only stimulation at some doses of Pb. The analysis of enzymatic activity in paddy and corn fields polluted with Pb, Cd, Cu, and Zn indicated that HMs inhibited cellulolytic activity in more polluted paddy fields [38]. In the presence of Cd, five epiphytes (except for *Agrobacterium* sp. AzoEpi18) and *Acinetobacter* sp. AzoEndo8 developed larger halos than in the control, while the other strains showed only partial stimulation at several doses. *Achromobacter* sp. AzoEpi1, *Bacillus* sp. AzoEpi2, *Microbacterium* sp. AzoEpi6, and *Delftia* sp. AzoEpi7 and *Acinetobacter* sp. AzoEndo8 exhibited stronger cellulolytic degradation in the presence of Cr (VI), whereas higher degradation by *Alcaligenes* sp. AzoEpi21 was recorded only at the higher doses of this metal. The activity of the three other endophytes and *Agrobacterium* sp. AzoEpi18 was reduced at the exposure to Cr. Aslam et al. [17] demonstrated inhibition of cellulase activity of *Bacillus amyloliquefaciens*-ASK11 during exposure to Cr (VI). This response was strongly related to the Cr-dose: the activity was 2 times lower at 50 mg L^−1^, 4 times lower at 100 mg L^−1^ and 10 times lower at 250 mg L^−1^ and higher doses than in the untreated control [17]. In our study, the endophytic *Bacillus* sp. exhibited lower CMC-activity in the presence of Cr, but the epiphytic strain showed increased production of the enzyme in comparison to the control. Ni had a more negative effect on cellulose degradation, as only *Achromobacter* sp. AzoEpi1 and *Bacillus* sp. AzoEpi2 showed increased activity in the whole range of the metal concentration, *Microbacterium* sp. AzoEpi6, *Delftia* sp. AzoEpi7, and *Alcaligenes* sp. AzoEpi21 showed partial stimulation, and the four other strains displayed lower cellulolytic activity than in the control. The noble metals had strong negative effects on enzyme activity. Only *Microbacterium* sp. AzoEpi6 and *Delftia* sp. AzoEpi7 were stimulated to degrade more cellulose in the Ag (both strains) and Au treatments (*Delftia* sp.).

In general, the xylanolytic activity was reduced in most cases, and Cd and Cr had the strongest effects, affecting all strains except for *Bacillus* sp. AzoEndo3 (0.5–1 mg Cd L^−1^) and *Alcaligenes* sp. AzoEpi21 (50 mg Cr L^−1^). In the case of Pb, lower activity was exhibited by *Microbacterium* sp. AzoEpi6, *Delftia* sp. AzoEpi7, and *Alcaligenes* sp. AzoEpi21, as well as *Bacillus* sp. AzoEndo3 and *Staphylococcus* sp. AzoEndo11, while the other strains showed partial potential for enhanced xylan degradation: *Achromobacter* sp. AzoEpi1, *Bacillus* sp. AzoEpi2, *Agrobacterium* sp. AzoEpi18, *Acinetobacter* sp. *Micrococcus* sp. AzoEndo7, and *Acinetobacter* sp. AzoEndo8—100–500 mg Pb L^−1^. The presence of Ni led to reduction of xylanase activity of five strains (*Microbacterium* sp. AzoEpi6, *Delftia* sp. AzoEpi7, *Agrobacterium* sp. AzoEpi18, *Bacillus* sp. AzoEndo3, and *Staphylococcus* sp. AzoEndo11), while *Achromobacter* sp. AzoEpi1 and *Bacillus* sp. AzoEpi2 exhibited stimulation of the activity. The other three strains, namely *Alcaligenes* sp. AzoEpi21, *Micrococcus* sp. AzoEndo7, and *Acinetobacter* sp. AzoEndo8, showed increased degradation at 100 mg Ni L^−1^ (epiphyte) and 50 mg Ni L^−1^ (both endophytes). In the case of the Ag treatments, 6 out of the 10 strains (*Agrobacterium* sp. AzoEpi18, *Alcaligenes* sp. AzoEpi21 and all endophytes) exhibited lower xylanase activity in comparison to the control. The exposure to Au revealed that two strains (*Achromobacter* sp. AzoEpi1 and *Delftia* sp. AzoEpi7) had higher activity in the whole range of doses, and *Bacillus* sp. AzoEpi2 degraded greater amounts of xylan at 5 mg Au L^−1^.

The overall analysis of proteolytic activities revealed the predominance of inhibiting effects of the tested metals. Only *Alcaligenes* sp. AzoEpi21 was able to degrade more proteins than in the control in the whole range of Cr (VI). In the treatment with this metal, AzoEpi1, AzoEpi7, and AzoEpi18 showed higher proteolytic potential at 50 mg Cr L^−1^. Kheirabadi and co-workers [39] studied the ability of *Shewanella* sp. to remove Cr (VI). They found that the protease activity was very high (>140 mg mL^−1^) at 50 mg Cr L^−1^ and 2–3 times lower at a higher concentration of the metal. The authors suggested that the presence of serine protease helps *Shewanella* sp. to cope with the metal. Ag had the most negative inhibitory effects on proteases in all strains. In the case of Au, only *Microbacterium* sp. AzoEpi6 was able to decompose greater amounts of proteins at 5 mg Au L^−1^. In the Ni treatment, only *Achromobacter* sp. AzoEpi1 and *Microbacterium* sp. AzoEpi6 as well as *Micrococcus* sp. AzoEndo7 and *Acinetobacter* sp. AzoEndo8 showed protease activity at 50 mg Ni L^−1^. During the exposure to Pb, five strains were able to decompose proteins partially (doses of 100–500 mg L^−1^) at higher amounts than in the control. These strains were all endophytes, and epiphytic *Achromobacter* sp. Cd was the only treatment in which all strains showed partial potential to produce more proteases ranging between 0.5 and 5 mg Cd L^−1^, depending on the strain. Li and co-workers [38] reported a stimulating effect of HMs on proteolytic activity. They concluded that such an effect was related to the presence of a higher pool of proteins derived from the degradation processes carried out by soil microorganisms.

## 5. Conclusions

Our results suggest that the bacterial isolates can have dual functions as a bioremediation agent and a potential plant growth promoting biofertilizer.

*Delftia* sp. AzoEpi7 displayed the highest phenotype change in our study—it was the most efficient in metabolizing carboxylic acids, surfactants (including persistent Niaproof 4), dyes, and other organic compounds and had the highest cellulolytic activity in the presence of five metals (Pb, Cd, Cr, Ag and Au). *Staphylococcus* sp. AzoEndo11 responded to three factors—it grew in the widest range of pH and efficiently metabolized antibiotics and inorganic compounds. *Achromobacter* sp. AzoEpi1 exhibited the highest xylanolytic and proteolytic potential in the Ni, Ag, and Au as well as Pb, Cd and Ni treatments, respectively, and very high tolerance to HMs—all doses except for 10 mg Au L^−1^. *Microbacterium* sp. AzoEpi6 were characterized by the strongest metabolizing potential for carbohydrates and drugs, including N-acetylneuraminic acid, which was difficult to degrade by the other strains. *Agrobacterium* sp. AzoEpi18 showed the highest tolerance to the tested metals (4 doses of Pb and all doses of Cd, Cr, Ni, Ag, and Au) and degradation of amino acids. The fourth group comprised *Alcaligenes* sp. AzoEpi21 showing the highest tolerance to salinity stress and *Micrococcus* sp. AzoEndo7 with the highest changes in IAA during exposure to Cr, Ni, Ag, and Au. Both *Bacillus* isolates and *Acinetobacter* sp. AzoEndo8 showed hardly any phenotype change during the exposure to the analyzed factors. Moreover, it should be noted that each strain has individual tolerance to the tested parameters or potential to metabolize specific compounds, which makes all strains beneficial as both biofertilizers and bioremediation agents, especially when combined in one biopreparation.

## Figures and Tables

**Table 1:** Strains selected for the study and their accession numbers from the GenBank database [9].

Strain Name	Code	GenBank NO.
*Achromobacter* sp. AzoEpi1	AzoEpi1	MG881884
*Bacillus* sp. AzoEpi2	AzoEpi2	MG881885
*Microbacterium* sp. AzoEpi6	AzoEpi6	MG881889
*Delftia* sp. AzoEpi7	AzoEpi7	MG881890
*Agrobacterium* sp. AzoEpi18	AzoEpi18	MG881901
*Alcaligenes* sp. AzoEpi21	AzoEpi21	MG881904
*Staphylococcus* sp. AzoEndo11	AzoEndo11	MH605511
*Micrococcus* sp. AzoEndo7	AzoEndo7	MG881917
*Bacillus* sp. AzoEndo3	AzoEndo3	MG859254
*Acinetobacter* sp. AzoEndo8	AzoEndo8	MG881918

AzoEpi indicates an epiphyte and AzoEndo denoted an endophyte.

**Table 2:** MIC for each treatment.

Strain	Pb	Cd	Cr	Ni	Ag	Au
*Achromobacter* sp. AzoEpi1	1000	10	100	100	10	5
*Bacillus* sp. AzoEpi2	500	5	100	50	10	5
*Microbacterium* sp. AzoEpi6	1000	10	100	50	10	5
*Delftia* sp. AzoEpi7	200	0.5	100	100	10	10
*Agrobacterium* sp. AzoEpi18	500	10	100	100	10	10
*Alcaligenes* sp. AzoEpi21	200	10	100	100	10	5
*Staphylococcus* sp. AzoEndo11	500	10	50	50	10	5
*Micrococcus* sp. AzoEndo7	500	5	50	50	10	5
*Bacillus* sp. AzoEndo3	1000	5	100	50	10	5
*Acinetobacter* sp. AzoEndo8	1000	5	100	100	10	5

## Data Availability

Data are contained within the article or supplementary material.

## Supplementary Materials

The following are available online at www.mdpi.com/article/10.3390/biology10090879/s1, Supplementary Figure S1. Effect of the metals applied in the specified range of doses (in mg L^−1^) on the cellulolytic activities of the selected strains expressed as differences in the size of halos (in mm) vs. the untreated control. Positive values of bars—stimulation of the activity; negative values of bars—inhibition of the activity. See Table 1 for strain codes, Supplementary Figure S2. Effect of the metals applied in the specified range of doses (in mg L^−1^) on the xylanolytic activities of the selected strains expressed as differences in the size of halos (in mm) vs. the untreated control. Positive values of bars—stimulation of the activity; negative values of bars—inhibition of the activity. See Table 1 for strain codes, Supplementary Figure S3. Effect of the metals applied in the specified range of doses (in mg L^−1^) on the proteolytic activities of the selected strains expressed as differences in the size of halos (in mm) vs. the untreated control. Positive values of bars—stimulation of the activity; negative values of bars—inhibition of the activity. See Table 1 for strain codes.
