# Supplementary material for: Phenotype Switching in Metal-Tolerant Bacteria Isolated from a Hyperaccumulator Plant
Source: Biology (Basel). 2021 Sep 7;10(9):879. doi: 10.3390/biology10090879 (PMC8466758; doi:10.3390/biology10090879)

Supplementary Figure S1. Effect of the metals applied in the specified range of doses (in mg L^{-1}) on the cellulolytic activities of the selected strains expressed as differences in the size of halos (in mm) vs. the untreated control. Positive values of bars - stimulation of the activity; negative values of bars - inhibition of the activity. See Table 1 for strain codes.

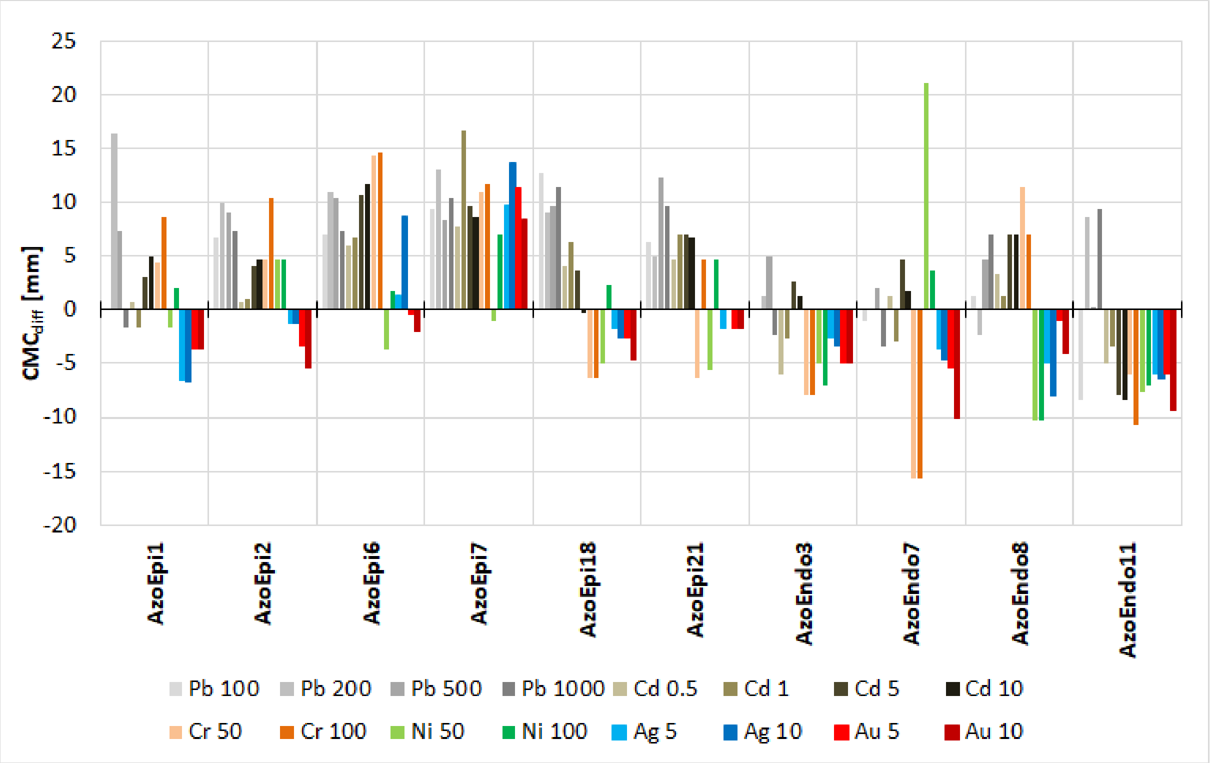

Supplementary Figure S2. Effect of the metals applied in the specified range of doses (in mg L^{-1}) on the xylanolytic activities of the selected strains expressed as differences in the size of halos (in mm) vs. the untreated control. Positive values of bars - stimulation of the activity; negative values of bars - inhibition of the activity. See Table 1 for strain codes.

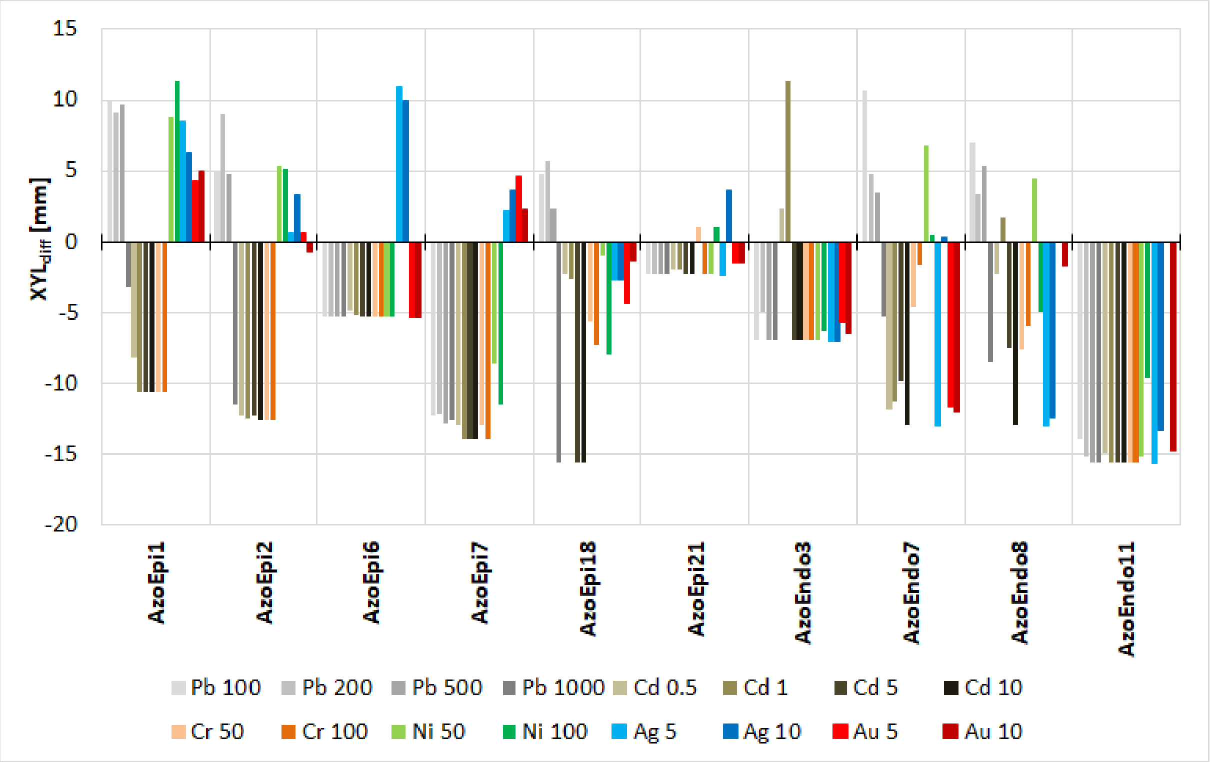

Supplementary Figure S3. Effect of the metals applied in the specified range of doses (in mg L^{-1}) on the proteolytic activities of the selected strains expressed as differences in the size of halos (in mm) vs. the untreated control. Positive values of bars - stimulation of the activity; negative values of bars - inhibition of the activity. See Table 1 for strain codes.

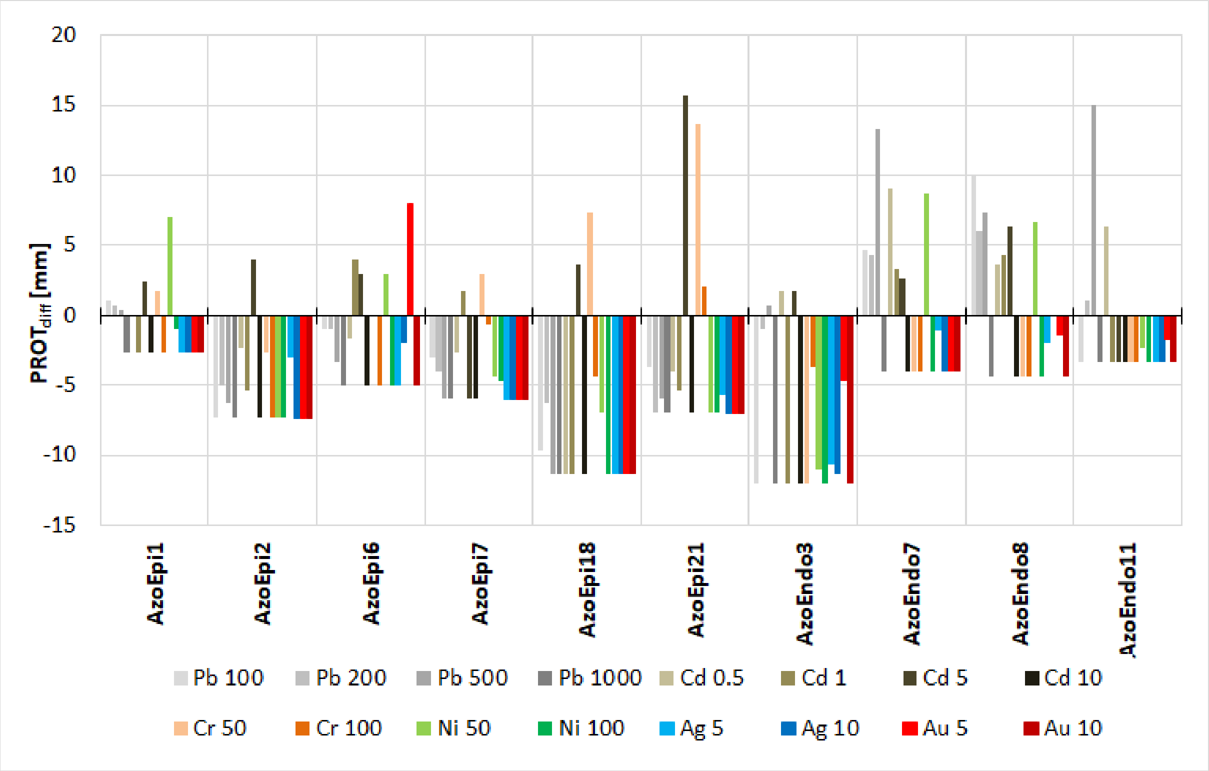

Supplement: Supplementary file 1 [file biology-10-00879-s001.zip › biology-1324601-supplementary.pdf]
